# *Toxoplasma gondii* Migration within and Infection of Human Retina

**DOI:** 10.1371/journal.pone.0054358

**Published:** 2013-02-21

**Authors:** João M. Furtado, Liam M. Ashander, Kathleen Mohs, Timothy J. Chipps, Binoy Appukuttan, Justine R. Smith

**Affiliations:** 1 Casey Eye Institute, Oregon Health & Science University, Portland, Oregon, United States of America; 2 Department of Cell and Developmental Biology, Oregon Health & Science University, Portland, Oregon, United States of America; Albert Einstein College of Medicine, United States of America

## Abstract

Toxoplasmic retinochoroiditis is a common blinding retinal infection caused by the parasite, *Toxoplasma gondii*. Basic processes relating to establishment of infection in the human eye by *T. gondii* tachyzoites have not been investigated. To evaluate the ability of tachyzoites to navigate the human retina, we developed an *ex vivo* assay, in which a suspension containing 1.5×10^7^ parasites replaced vitreous in a posterior eyecup. After 8 hours, the retina was formalin-fixed and paraffin-embedded, and sections were immunostained to identify tachyzoites. To determine the preference of tachyzoites for human retinal neuronal versus glial populations, we infected dissociated retinal cultures, subsequently characterized by neuron-specific enolase or glial fibrillary acidic protein expression, and retinal cell lines, with YFP-expressing tachyzoites. In migration assays, retinas contained 110–250 live tachyzoites; 64.5–95.2% (mean  = 79.6%) were localized to the nerve fiber layer, but some were detected in the outer retina. Epifluorescence imaging of dissociated retinal cultures 24 hours after infection indicated preferential infection of glia. This observation was confirmed in growth assays, with significantly higher (p≤0.005) numbers of tachyzoites measured in glial verus neuronal cell lines. Our translational studies indicate that, after entering retina, tachyzoites may navigate multiple tissue layers. Tachyzoites preferentially infect glial cells, which exist throughout the retina. These properties may contribute to the success of *T. gondii* as a human pathogen.

## Introduction

Ocular toxoplasmosis is a common inflammatory eye disease that may complicate systemic infection with the parasite, *Toxoplasma gondii* [1]. Approximately 1.25 million U.S. residents suffer from ocular toxoplasmosis, and in certain parts of the world this eye disease is believed to affect over 15% of the population [2]. While the devastating effects on vision in immunocompromised patients and unborn children are often emphasized, it is important to recognize that numerically, the largest group seeking treatment for ocular toxoplasmosis consists of otherwise healthy adults [3]. When infecting immunocompetent persons, parasites lodge in the eye and other parts of the central nervous system, and in skeletal and cardiac muscle [4]. Clinical manifestations usually result from infection of the retina, with secondary involvement of the choroid in the most severe cases [5]. Accordingly, ocular toxoplasmosis is more specifically termed ‘toxoplasmic retinochoroiditis’. Standard antimicrobial treatments may suppress growth of the parasite, but side effects are common and none of the available drugs achieve eradication of encysted parasites from the retina [6].

During the past 10 years, genetic manipulation of the parasite genome has rapidly advanced our knowledge of *T. gondii* pathobiology. Our understanding of the general immunological mechanisms responsible for host-parasite recognition and the host immune response to the parasite also has advanced considerably. On the other hand, relatively little attention has been focused on the interaction of the host and *T. gondii* in toxoplasmic retinochoroiditis. Information on this subject has been generated largely in experiments using mice. Yet, considerable differences exist between human and murine responses to *T. gondii*. Many basic questions relating to the establishment of an ocular infection in the human eye have not been addressed. One important question that lacks an answer relates to the establishment of infection in the retina: after entering the human retina, does the parasite migrate within the retina and which retinal cell subtypes does the parasite infect preferentially? The studies described here show that tachyzoites are able to migrate through human retina and grow in supporting glial cells in preference to neurons.

## Materials and Methods

### Parasites

RH strain *T. gondii* were maintained in tachyzoite form by serial passage in confluent monolayers of human foreskin fibroblasts in Dulbecco's modified Eagle's medium (DMEM; Catalogue number: 12100; Invitrogen-Gibco, Grand Island, NY), supplemented with 44 mM sodium bicarbonate and 1% heat-inactivated fetal bovine serum (FBS; Hyclone, Logan, UT), at 37°C and at 5% CO2. For some assays, a transgenic RH strain with a plasmid that contained a tandem-repeat yellow fluorescent protein (YFP) under control of the SAG1 promoter (kind gift of Boris Striepen, PhD, University of Georgia, Athens, GA) [7] was used. Plaque assays were performed using a fibroblast monolayer to indicate parasite viability at the time of each experiment. Experiments were included only when parasite viability was at least 35%, consistent with published measurements [8].

### Human eyes

Posterior eyecups were obtained from male and female cadaveric donors with no history of retinal disease via Lions VisionGift (Portland, OR). Three donors used for the intraretinal migration assays were aged 57 to 75 years at the time of death, and the experiment commenced within 50 hours of death. Three donors used as the source for dissociated retinal cultures were aged 45 to 59 years at time of death, and death-to-culture time was 24 hours or less.

### Cell lines

Human foreskin fibroblasts (Cascade Biologics, Portland, OR), MIO-M1 human Müller glial cells (gift of G. Astrid Limb, PhD, and Peng T. Khaw, MD, PhD, University College London, London, United Kingdom) [9] and Y79 human retinoblastoma cells (American Type Culture Collection (ATCC), Manassas, VA) were used in some experiments. Y79 cells naturally grow in suspension, but were induced to adhere to culture surfaces following the method of McFall et al., [10] which involved pre-coating surfaces with 1 mg/ml poly-l-ornithine HBr (Sigma-Aldrich, St. Louis, MO) in phosphate buffered saline (PBS) for 18 hours at room temperature, followed by 2 washes with PBS plus 0.1% ethylenediaminetetraacetic acid (EDTA; Invitrogen, Carlsbad, CA) and 2 subsequent washes with Roswell Park Memorial Institute medium (RPMI) 1640 (ATCC) supplemented with 20% FBS.

### Intraretinal *T. gondii* migration assay

Right and left posterior eyecups were positioned on sheets of Styrofoam using 30G needles placed through conjunctiva and external ocular muscle attachments, with optic nerve located posteriorly. Lens and iris were dissected carefully from each eyecup using forceps and scissors. To provide a clear visualization of the retina, up to 6 5–0 monofilament nylon sutures (Supramid Extra; S. Jackson Inc., Alexandria, VA) were placed through the eye wall along the margin of the eyecup at the level of the ciliary body under a surgical operating microscope. Vitreous was removed as completely as possible from the eyecups, taking care not to tear the retina. Initially, the tissue was disengaged using scissors. As a result, the majority of vitreous was readily expelled, by simply tipping the eyecup. Remaining vitreous was removed by isolating adherent strands with a cotton-tipped stick and excising these strands with scissors. Empty posterior eyecups were placed in wells of a 12-well plate with the optic nerve facing posteriorly, and filled with 1.5 ml of a suspension of freshly egressed tachyzoites in supplemented DMEM (1.0×10^7^ tachyzoites/ml). Tachyzoites were not delivered to the posterior eyecup in leukocytes, in order to limit the duration of the experiment to a period during which the retina would remain viable, and to allow for delivery of all parasites simultaneously to the eyecup. The suspension applied to the right eyecup contained viable tachyzoites, and the suspension applied to the left eyecup contained heat-killed tachyzoites (i.e., incubated in a 55°C water bath for 1 hour). Both eyecups were incubated at 37°C and 5% CO_2_ for 8 hours. After the incubation, suspensions of tachyzoite were removed, and the cavity of each eyecup was washed 6 times with PBS. Eyecups were flattened by placing 4 equally spaced radial incisions with scissors and fixing the whole-mounts to sheets of Styrofoam, as described above. Central neural retina was excised from each eyecup using a 16 mm corneal trephine (Katena, Denville, NJ), and retina was separated from the optic nerve head with scissors.

### Detection of migrated *T. gondii* in human retina

Central retina was fixed for 4 hours in 10% neutral buffered formalin, followed by 70% ethanol for a minimum of 24 hours, and subsequently embedded in paraffin. The tissue was sectioned at 5 µm thickness and mounted on SuperFrost Plus glass slides (Fisher Scientific, Santa Clara, CA). Before staining, slides were baked for approximately 1 hour at 58°C. Detection of tachyzoites was performed by indirect immunohistochemistry, following a previously published method, [11] and using mouse anti-SAG1 monoclonal antibody, DG52 (kind gift of Dr. L. David Sibley, Washington University, St. Louis, MI), [12] at a concentration of 2 µg/ml. Antigen retrieval was not required. Antibody-antigen complexes were visualized in the tissue with Fast Red. Hematoxylin counterstaining was performed to highlight retinal anatomy. Stained sections were mounted with Clear-Mount with Tris Buffer (Electron Microscopy Sciences, Hatfield, PA). All tissue samples were sectioned in entirety, and for a conservative quantification of tachyzoite number with no parasite counted more than once, every fifth section was stained with anti-SAG1 antibody. For negative controls, an equivalent number of intervening sections were stained using 2 µg/ml mouse immunoglobulin (Ig)G (Vector Laboratories, Burlingame, CA) in place of specific primary antibody. Sections were viewed by light microscopy at 100× and 400× magnification, and tachyzoites were counted and localized to individual retinal layers. Tachyzoites located along the external limiting membrane or at tissue margins were excluded. Removal of the neural retina from an unfixed posterior eyecup results in disruption of the photoreceptor layer. Since this layer was not uniformly intact, tachyzoites in this location were not included in the quantification.

### *T. gondii* infection of dissociated human retinal cultures

Preparation of human retinal cultures was performed following a method modified from Gaudin et al [13]. After removal of lens and vitreous from posterior eyecups, retina was teased free from choroid. The retina was gently washed in warm DMEM supplemented with 44 mM sodium bicarbonate, to remove any adherent vitreous and retinal pigment epithelium, divided into pieces measuring approximately 2×2 mm, and incubated in modified DMEM containing 2 mg/ml papain (Sigma-Aldrich) with frequent swirling at 37°C and in 5% CO_2_. After 30 minutes, 20% FBS was used to arrest digestion. Tissue was processed further by addition of 0.1 mg/ml DNase I (Sigma-Aldrich) and trituration. Large debris was allowed to settle, and the supernatant was centrifuged at 120×*g* for 5 minutes. Pelleted cells were suspended in 1∶1 modified DMEM:Hams F12 medium (Hyclone) with 2% FBS, and 50 U/ml penicillin and 50 μg/ml streptomycin (both from Invitrogen-Gibco), and plated at 2×10^6^ cells in 35 mm Fluorodish glass-bottom dishes (World Precision Instruments, Sarasota, FL) that had been serially pre-coated with 0.1 μg/ml poly-l-lysine and 0.5 mg/ml human laminin (both from Sigma-Aldrich) for 15 minutes each and dried for 2 hours at 37°C. Cultures remained undisturbed at 37°C and in 5% CO_2_ for 5 days, after which time medium was changed every 3 to 4 days. After approximately 5 to 6 weeks, when cell cultures presented a mat of retinal glial cells supporting retinal neurons, they were exposed to 1×10^6^ freshly egressed YFP-expressing *T. gondii* tachyzoites suspended in serum-free 1∶1 modified DMEM:Hams F12 medium for 24 hours.

### Immunostaining of *T. gondii*-infected dissociated human retinal cultures

Dishes containing infected dissociated human retinal cultures were washed twice with PBS, and cells were fixed in 4% paraformaldehyde for 5 minutes, and blocked and permeabilized by treatment with 3% normal donkey serum (Sigma-Aldrich) and 0.05% triton X-100 in PBS for 15 minutes at room temperature. Cultures were stained for 2 hours at room temperature with 1∶500 rabbit polyclonal anti-human neuron-specific enolase (NSE; Polysciences Inc., Warrenton, PA) or 10 ug/ml sheep polyclonal anti-human glial fibrillary acidic protein (GFAP; R&D Systems), both diluted in the blocking solution. Control cultures were stained with normal rabbit serum (Sigma-Aldrich) or sheep IgG (Sigma-Aldrich), similarly diluted, in place of specific primary antibodies. Dishes were washed twice in PBS, and subsequently cultures were incubated for 1 hour at room temperature with Alexa Fluor 594-conjugated donkey anti-rabbit or donkey anti-sheep secondary antibodies (Invitrogen-Molecular Probes, Eugene, OR), diluted in blocking solution to 5 ug/ml. After 2 washes with PBS, cultures were fixed in 4% paraformaldehyde for an additional 10 minutes before mounting with Fluoromount-G (SouthernBiotech, Birmingham, AL). The immunostained cultures were imaged at 630X magnification by bright field microscopy, and by epifluorescence microscopy with YFP and red fluorescent protein filters.

### *T. gondii* infection of human retinal cell lines

We used the “High Throughput Growth Assay for *Toxoplasma gondii*” described by Gubbels et al. [7] to compare *T. gondii* tachyzoite infection of retinal neurons versus retinal glial cells. MIO-M1 human Müller glial cells, Y79 retinoblastoma cells and positive control human foreskin fibroblasts were plated in 96-well plates and allowed to grow to confluence over a 5-day period in DMEM with 10% FBS (MIO-M1 and fibroblasts) or RPMI 1640 with 20% FBS (Y79) at 37°C and 5% CO_2_. Cell numbers at seeding were 7,500/well for MIO-M1, 60,000/well for Y79 and 5,000/well for fibroblasts. Wells of each cell population (n = 7–8 wells/condition) were infected with 1×10^5^ freshly egressed YFP-expressing *T. gondii* tachyzoites suspended in serum and phenol red-free DMEM (MIO-M1 and fibroblasts) or RPMI 1640 with 1 mM sodium pyruvate and 10 mM HEPES (Y79 cell). Cells were not washed in order to avoid disruption of the cell monolayers; a preliminary study established the number of parasites required to ensure that the fluorescence of a freshly infected cell monolayer was no greater than the background fluorescence of uninfected cultures. Control wells were filled with the same medium, but without tachyzoites. Plates were incubated for 48 hours at 37°C and 5% CO_2_, and increase in fluorescence of wells between 24 and 48 hours post-infection was measured as an indicator of parasite growth on the Victor3 Multilabel Counter 1420 (PerkinElmer, Waltham, MA). Background fluorescence was determined as fluorescence of control wells containing the same cells at the same time points.

### Statistical analyses

For statistical analyses, the Student's t-test, two-tailed, was used to compare results obtained under two different conditions. In these comparisons, a p value less than 0.05 was taken to indicate a statistically significant difference.

## Results

### *T. gondii* tachyzoites migrate through human retina

**Figure 1:**
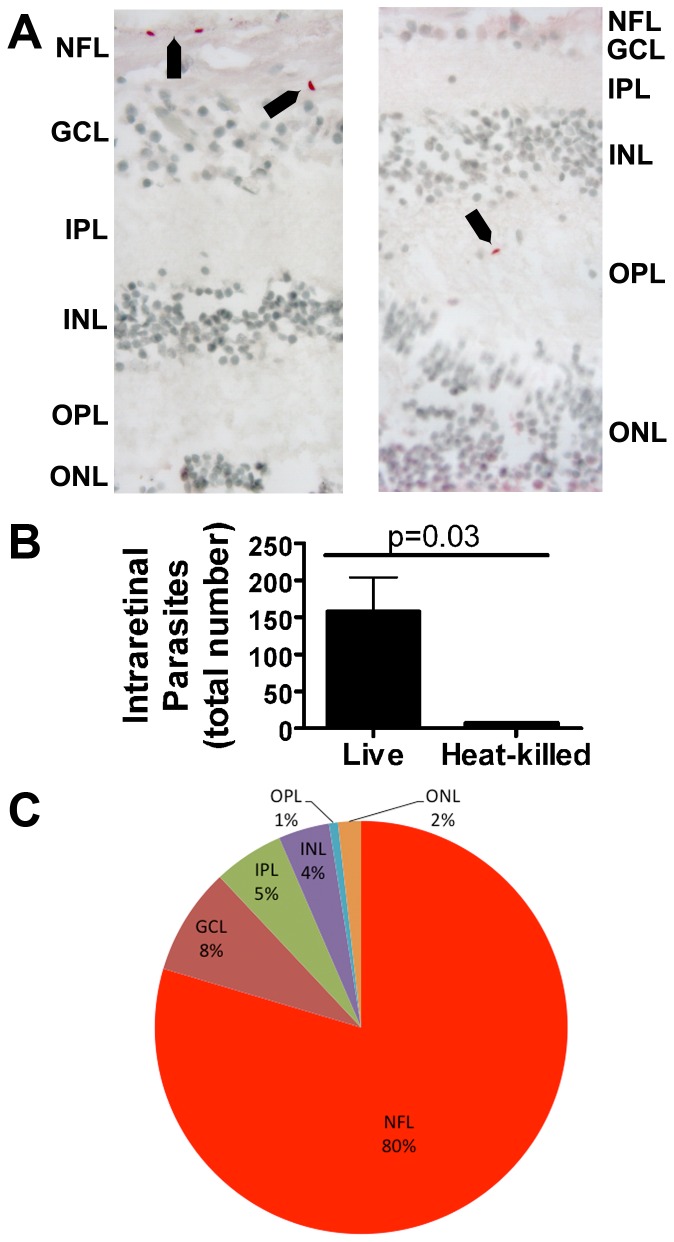
*T. gondii* tachyzoites migrate through human retina. (A). Representative photomicrographs showing *T. gondii* tachyzoites within the retina 8 hours following addition of a suspension of 1.5×10^7^ live parasites to a human posterior eyecup. Tachyzoites are identified by immunostaining for the parasite SAG-1 antigen. Fast Red with hematoxylin counter stain. Original magnification: 1000X. Arrows indicate tachyzoites. Negative control sections showed no positive staining. (B). Graph showing number of tachyzoites counted within retinas from eyecups incubated with live or heat-killed tachyzoites. Columns  =  mean. Error bars  =  standard error of mean. (C). Pie chart showing mean percentage of tachyzoites located at different retinal layers. NFL  =  nerve fiber layer; GCL  =  ganglion cell layer; IPL  =  inner plexiform layer; INL  =  inner nuclear layer; OPL  =  outer nuclear layer; ONL  =  outer nuclear layer. Data shown in (B) and (C) were generated in experiments using 3 paired human cadaver posterior eyecups.

To evaluate the ability of *T. gondii* tachyzoites to migrate through human retina, we developed an *ex vivo* assay, in which a suspension containing 1.5×10^7^ tachyzoites replaced the vitreous in an intact posterior eyecup. The assay did not incorporate parasite migration from within the retinal vessels into the retina, as would occur *in vivo*, which is not possible in a human-based experimental system. However, since the retinal vessels ramify in the nerve fiber layer and their branches are confined to the inner retina, [14] application of parasites at the level of the inner limiting membrane resembles the arrival of parasites in the retina *in vivo* as closely as possible. For 3 different human cadaver donors, live tachyzoites were applied to right posterior eyecups, and heat-killed tachyzoites were applied to left posterior eyecups. After an 8-hour incubation, the retinal tissue appeared grossly and histologically viable. In immunostained ocular sections from retinas incubated with live tachyzoites (Figure 1A), we identified 110–250 (mean  = 158.0) parasites within the retinal layers per eyecup (Figure 1B). Most intraretinal parasites (64.5–95.2%, mean  = 79.6%) were located at the nerve fiber layer, but we also detected parasites in deeper layers: ganglion cell layer (2.0–14.0%, mean  = 8.4%); inner plexiform layer (2.0–10.9%, mean  = 5.5%); inner nuclear layer (0.4–10.9%, mean  = 4.0%); outer plexiform layer (0–1.6%, mean  = 0.7%); and outer nuclear layer (0–4.5%, mean  = 1.8%) (Figure 1C). No evidence of tachyzoite replication was observed within the retina. We also observed a small number of parasites in sections of eyecups incubated with heat-killed tachyzoites, i.e., 5–9 (mean  = 7.3) parasites per eyecup, which we attributed to processing artifact. Negative control sections immunostained with mouse IgG in place of specific primary antibody showed no positive staining.

### *T. gondii* tachyzoites infect human retinal glial cells in preference to neurons

**Figure 2:**
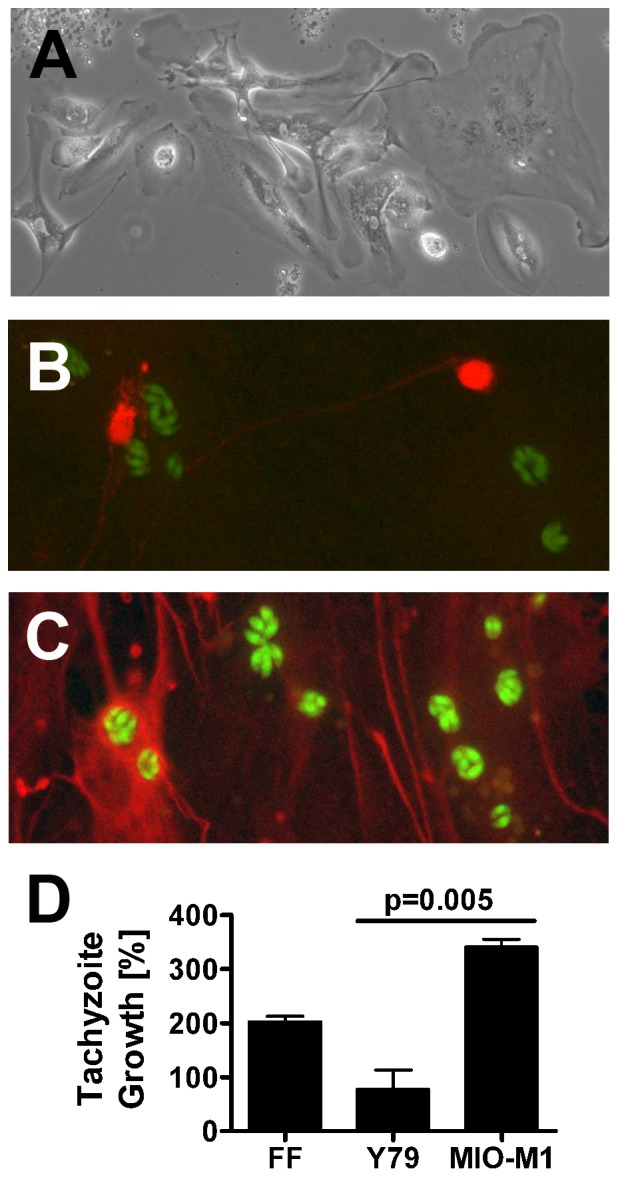
*T. gondii* tachyzoites infect human retinal glial cells in preference to neurons. (A). Immediately prior to infection, dissociated human retinal cultures presented a layer of glial cells with neurons positioned above. Original magnification: 100X. (B and C). Expression of (B) neuron specific enolase (NSE) (red), as detected by rabbit polyclonal anti-human NSE antibody and Alexa Fluor 594-conjugated donkey anti-rabbit immunoglobulin (Ig)G antibody and (C) glial fibrillary acidic protein (GFAP) (red), as detected by sheep polyclonal anti-human GFAP antibody and Alexa Fluor 594-conjugated donkey anti-sheep IgG antibody. *T. gondii* tachyzoites express YFP (green). Original magnification: 630X. Negative control cultures showed no positive staining. (D). Graph showing percentage growth of tachyzoites in Y79 human retinoblastoma cells and MIO-M1 human Müller glial cells, plus positive control human foreskin fibroblasts (FF), over a 24-hour period. n = 7–8 wells/condition. Columns  =  mean. Error bars  =  standard error of mean. Representative of two independent experiments.

To clarify whether *T. gondii* tachyzoites showed preferential growth in human retinal neuronal versus glial subpopulations, we prepared dissociated human retinal cultures. As described in publications from the laboratory of David Hicks, PhD (Institut national de la santé et de la recherche médicale, Strasbourg, France), where the methodology was developed and the cultures were characterized in detail, [13], [15] we observed human dissociated retina to exist for multiple weeks as a layer of glial cells supporting scattered neurons in variable numbers. Between 34 and 43 days (Figure 2A), the cultures were exposed to 1×10^6^ YFP-expressing *T. gondii* tachyzoites for 24 hours and subsequently stained to detect either human NSE (marker of neurons) or human GFAP (marker of glial cells). Epifluoresent visualization of the stained cultures suggested that retinal glial cells more readily supported the growth of tachyzoites in comparison to retinal neurons (Figures 2B and 2C). However, due to the difference in cell numbers, morphology and growth pattern, this observation could not be quantitated. Negative control cultures showed no specific staining. The presence of SAG-1 transcript, but not BAG-1 transcript, by RT-PCR on RNA from infected dissociated human retina, implied stage conversion was not occurring during the course of the experiment (data not shown).

Since studies using dissociated human retina generated qualitative results, but did not provide a quantitative comparison of parasite growth in retinal neuronal versus glial cell populations, we used a published assay to compare behavior of tachyzoites in human retinal Y79 neuronal and MIO-M1 glial cell lines. Growth within human fibroblasts was examined in parallel as a positive control for the infection. Confluent cell monolayers were simultaneously infected with YFP-expressing RH strain *T. gondii* tachyzoites. Intracellular tachyzoite growth was indicated by percentage increase in culture fluorescence, which was generated from readings obtained at 24 and 48 hours post-infection to capture the period of maximum growth ahead of cell lysis. After subtraction of mean background fluorescence for the same cell type, we observed a significantly greater increase in fluoresence of infected MIO-M1 versus Y79 cultures over the 24-hour period (340.6% versus 77.5%, p = 0.005; Figure 2D), supporting the previous observation that tachyzoites preferentially infected human retinal glial cells over retinal neurons. Microscopic observations at the end of the experiment revealed some loss of intactness of the monolayers. However, this disturbance was not greater for retinal neuronal cultures than for retinal glial cultures. Tachyzoites also proliferated rapidly within the positive control human fibroblast cultures. These findings were replicated in a second independent experiment.

## Discussion

Infection of the retina is the most common manifestation of toxoplasmosis in humans. Despite the common occurrence of toxoplasmic retinochoroiditis, basic processes that lead to the establishment of this infection have not been addressed. We have recently showed that the parasite may access the retina from the circulation in tachyzoite form, either unassisted [16] or in a leukocyte taxi [17]. Here we address the ability of *T. gondii* to move though human retina and to infect retinal cell populations after the eye is entered. We show that *T. gondii* tachyzoites are capable of moving through human retina, from the inner limiting membrane to the outer nuclear layer, in an *ex vivo* assay conducted in a human posterior eyecup. We also demonstrate qualitatively, using dissociated human retinal cultures, and quantitatively, using human retinal cell lines, that tachyzoites infect glial cells in preference to neurons in the human retina.

*T. gondii* tachyzoites invade host cells using a form of motility known as “gliding”, which is parasite actin-dependent [18]. The use of gliding to navigate biological barriers or to move through tissues has received considerably less attention, most likely because *T. gondii* is characterized as an intracellular parasite. However, it is clear that tachyzoites are able to cross epithelial and endothelial cell monolayers in vivo [16], [19]. Work conducted by Barragan and Sibley, [20] using an Ussing chamber, elegantly demonstrated the ability of tachyzoites to move through intact mouse ileum, across the epithelium and through the connective tissue of the lamina propria and submucosa. Our work shows that *T. gondii* is also capable of navigating a complex human tissue of clinical relevance. After crossing the blood-retinal barrier, tachyzoites encounter the highly complex neural retina, which includes three nuclear layers and three plexiform layers, as well as the photoreceptor processes. Our results indicate that over 90% of parasites move short distances and lodge in the nerve fiber layer and ganglion cell layer, which is consistent with separate clinicopathological reports describing two human eyes that were studied early in the course of infection when necrosis was localized to the inner retina [21], [22]. However, we also observed small numbers of tachyzoites reaching the outer retina. Our methods did not allow us to distinguish extracellular from intracellular parasites. A small proportion of all tachyzoites applied to the posterior eyecup entered the retina. This observation may reflect the *in vivo* situation. The result is probably also a function of the experimental system because: live parasite numbers decrease over time outside host cells; tachyzoites are required to cross the internal limiting membrane to enter the retina; and the assay is conducted over a short period of time to promote retinal viability.

Given that tachyzoites are able to navigate the human retina, they have access to various cell subtypes across its layers. Although it is clear that susceptibility to infection with *T. gondii* tachyzoites is dependent on cell type, [23]–[25] the preference of the parasite for different human retinal cell populations after it breaches the blood-retinal barrier has not been previously studied. Different *in vitro* studies indicate that tachyzoites may infect human Müller and retinal pigment epithelial cells, [26], [27] but do not address the issue of relative susceptibility, because tachyzoites can infect all nucleated cells [28]. Our results indicate that retinal neurons less readily support infection with tachyzoites than retinal glial cells. This difference in the course of infection in retinal glial cells and retinal neurons may relate to difference(s) in: (1) the ability of tachyzoites to invade each host cell population, which involves adhesion to the cell surface and penetration of the cell with formation of the parasitophorous vacuole from cell membrane; (2) the rate of tachyzoite division within each cell type; (3) the ability of tachyzoites to egress from each cell type; and (4) specific cellular response(s) to the infection. These stages of infection are impacted by cellular expression of various molecules, including host cell surface receptors and intracellular metabolites, as well as pro- and anti-microbial factors produced by the host cell in response to the infection [29]–[32]. Since retinal neurons are critical for sight, it is logical that these cells would exhibit properties less conducive to one or more stages of infection. Halonen et al. [33] reported that the percentage of infected cells was 2- to 3-fold higher and that replication of tachyzoites was 2- to 4-fold higher in human fetal cerebral astrocytes versus neurons exposed to *T. gondii*; extrapolating these results to retina suggests the possibility that retinal neurons are less readily invaded by tachyzoites and/or the rate of division within retinal neurons is relatively slow. Clarifying the mechanisms underlying the observed difference in *T. gondii* tachyzoite infection of retinal glial cells and neurons, as well as their molecular bases, will be an important area for future study.

In summary, we show in simple translational studies that *T. gondii* tachyzoites are capable of migrating through intact human retina, thus gaining access to multiple potential host cell populations. The tachyzoites preferentially grow within glial cells of the retina. Thus although retinal neurons resist infection, which might otherwise protect against retinal destruction, rapid tachyzoite growth in Müller cells ensures significant damage when the human retina becomes infected by *T. gondii*. These unique observations are relevant to the success of a human parasite, which infects approximately one-third of the world population.
